# Blood may buy goodwill: no evidence for a positive relationship between legal culling and poaching in Wisconsin

**DOI:** 10.1098/rspb.2017.0267

**Published:** 2017-11-22

**Authors:** Audun Stien

**Affiliations:** Norwegian Institute of Nature Research, Fram Centre, NO-9296 Tromsø, Norway

Chapron & Treves [1] tested the hypothesis that allowing some lethal legal control reduces the level of poaching. Using an analysis of data from the wolf populations in Michigan and Wisconsin, they discounted this hypothesis, concluding: … ‘allowing wolf (*Canis lupus*) culling was substantially more likely to increase poaching than reduce it’ [1, p. 1]. However, the text and analysis have in my opinion severe shortcomings, including: (i) biased reporting of previously published results, (ii) the use of a statistical model to evaluate density dependence in wolf area use that does not have theoretical or empirical support, and (iii) a failure to evaluate how between-year variations in reproductive rates affect their conclusions. When variation in reproductive rates is taken into account in their analysis, the conclusion is the opposite—allowing wolf culling is more likely to decrease poaching than increase it.

Chapron & Treves [1] cite, but seem to give a biased report of the findings from several published articles. They [1] claim that their analysis is the first to evaluate the relationship between legal culling and poaching in wolves. However, poaching of radio-tagged wolves has previously been shown to decrease in association with legal state culling in Wisconsin [2]. In the discussion they state: ‘As with prior studies on Wisconsin's wolf population [3], we did not detect any negative density dependence’ [1, p. 5]. However, the article they refer to does indeed find recruitment to be density-dependent. In addition, the occurrence of density dependence in wolf area use in Wisconsin is supported by a decrease in average wolf territory size as the population size increases [4]. Overall, it seems to me that results from the same study system that do not corroborate their own findings have been ignored when their own research is put in context.

Chapron & Treves [1] use a statistical model to evaluate density dependence in wolf area use in Wisconsin that has no theoretical or empirical support. They conclude that there is no evidence for density dependence in area use. However, when a more appropriate model is adopted, the data suggest weak evidence for density dependence. The model adopted by Chapron & Treves [1] was a log-linear model: 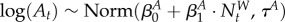, where log(*A_t_*) is the logarithm of the estimated total area used by wolves in Wisconsin in year *t*, 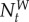 is the estimated total population size of wolves in Wisconsin in year *t* (based on interval censored data [1]), 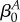 and 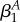 are regression coefficients estimated from the data, and *τ^A^* is an estimate of the residual variance in the data. They give no theoretical justification for their choice of model, or for their interpretation that a positive estimate of 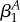 suggests no density dependence in area use. Furthermore, their model does not fit the available data well. The model overshoots the data at low and high population sizes while being below the data for mid population sizes (figure 1). In the case of no density dependence in wolf area use, the theoretical expectation is that the relationship between *A_t_* and 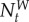 is linear: 
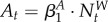
 (where 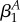 is the slope of the relationship and 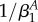 is the average density of wolves). A model adopted to evaluate evidence for density dependence in area use should preferably include this linear component, and a simple model that fulfils this criterion is the quadratic model: 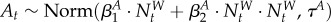. If the parameter 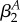 in this model is zero, the model describes a system where area use increases linearly with population size, i.e. wolf densities are constant independent of the population size. A negative estimate of 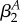 can be interpreted as evidence of negative density dependence, and suggests that the density of wolves increases with the population size. This quadratic model fits the available data well (figure 1), and the parameter estimates suggest weak density dependence in area use for the wolves in Wisconsin, as the estimated parameter for the quadratic relationship 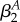 is significantly less than zero (
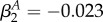
, s.e. = 0.008, 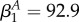, s.e. = 4.7; R script for all analyses in this Comment is found in the electronic supplementary material).

**Figure 1.**
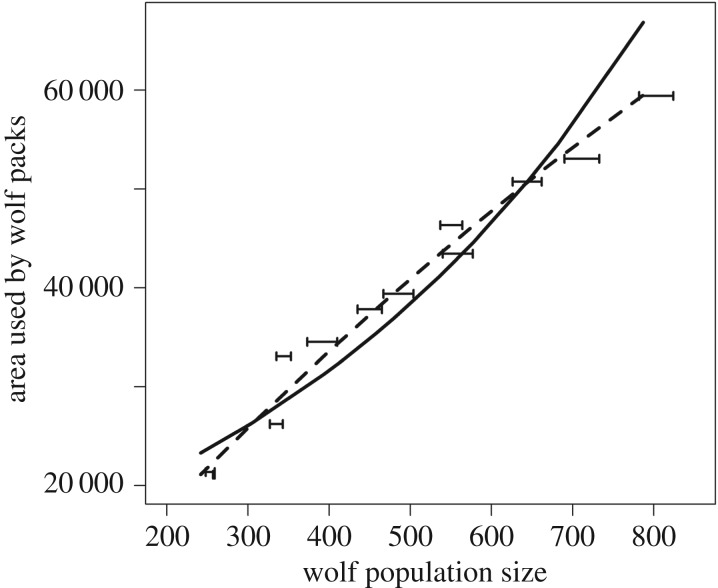
Area used by wolf packs in Wisconsin plotted against wolf population size. The minimum and maximum estimates of wolf population size are shown by the horizontal bars. The log-linear regression model fitted by Chapron & Treves [1] is plotted as a full line, while the quadratic model suggested in the text is plotted as a dotted line.

Chapron & Treves [1] explored the patterns in the between-year variation in reproductive rates to a minor degree by only evaluating the evidence for density dependence. However, a closer look at these data shows that the annual estimates of the probability for packs to reproduce decrease with legal state culling. The probability of reproduction was on average high in years with no legal state culling and lower in years when culling was legal for most of the year (figure 2, slope = −0.89, s.e. = 0.41, *p* = 0.03, binomial generalized linear mixed model with logit link function and year fitted as a random effect). This observed pattern in reproductive rates suggests that the tendency towards a negative relationship between legal state culling and population growth rates, interpreted as evidence of poaching by Chapron & Treves ([1], see also [5,6]), could alternatively be due to variation in reproductive rates. The temporal variation in reproductive rates could be owing to independent natural variation but may also have a causal component such as shooting of reproductive individuals during the legal cull [7]. To explore the role of variation in reproductive rates in explaining the negative relationship between population growth rates and legal state culling, I extended the main analysis in Chapron & Treves [1,8,9].

**Figure 2.**
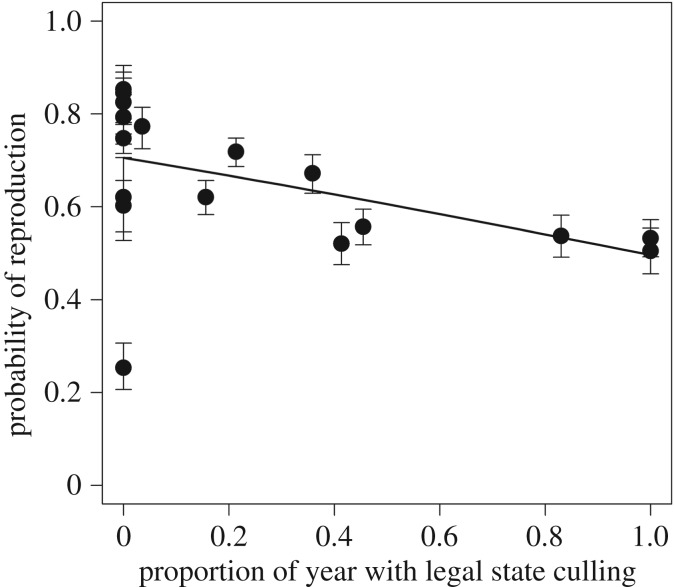
Annual estimates of the probability of reproduction in Wisconsin wolf packs (±1 s.e.) plotted against the proportion of the year with legal state culling. The regression line shows the best fit estimate from a generalized linear mixed model, with logit link function, a binomial error structure and year fitted as a random effect.

A first extension of their analysis revealed that the negative relationship between population growth rates and legal state culling is most evident in the data from Michigan, the state without supplementary data available for evaluation of alternative hypotheses. Chapron & Treves [1] modelled population growth rates in Wisconsin and Michigan as a linear function of the proportion of the year with legal state culling 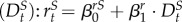, where 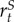 is the population growth rate in state *S* and year *t*. The model has a slope parameter for the impact of legal state culling (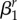) that is common for Michigan and Wisconsin. I fitted separate slope coefficients for the two states: 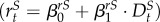. The parameter estimates of this model suggest that wolf population growth rates in Michigan show a stronger negative trend in relation to the period of legal state culling (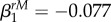, s.e. = 0.052) than those in Wisconsin (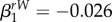, s.e. = 0.046).

In the next extension of the model, I included the probability of reproduction in Wisconsin 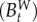
as a predictor of population growth rates in Wisconsin: 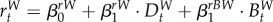. Not surprisingly, there was a strong positive relationship between the probability of reproduction in year *t* and the population growth rate from year *t* − 1 to *t* (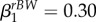, s.e. = 0.13). In this model, the estimated effect of legal state culling in Wisconsin tended to be positive (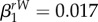, s.e. = 0.046) rather than negative. This shows that the tendency towards a negative relationship between legal state culling and population growth rates reported in [1] can be explained by the negative association between legal state culling and reproductive rates. Furthermore, if we interpret 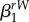 in this model as an estimate of the impact of legal state culling on poaching, the result lends support to the hypothesis that legal state culling reduces poaching, consistent with results from previous analyses of radio-tracked wolf survival in Wisconsin [2], and contrary to the conclusion in Chapron & Treves [1,6].

My conclusion is that there is negligible evidence for legal state culling to result in increased levels of poaching in these data [8,9]. This does not imply that poaching is not a problem in these states (e.g. [2]), but the conclusion that poaching increases with legal culling [1] is without empirical support. I acknowledge that there may be some degree of correlation between estimates of reproductive rates and population sizes owing to the study design. Such dependencies may inflate the estimates of the impact of reproduction on population growth rates. However, they cannot explain the observed negative relationship between estimates of legal state culling and reproductive rates (figure 2), and the pattern implies that variation in reproductive rates needs to be accounted for when interpreting changes in population growth rates as changes in poaching activity.

My analysis highlights the responsibility that researchers have to expose models to alternative hypotheses that are refined and biologically plausible. Such an approach is much more likely to improve understanding and allow a critical evaluation of different explanations. With respect to [1], this implies that variation in reproductive rates, as well as temporal variation in survival has to be considered when the pattern of variation in population growth rates is interpreted. Furthermore, it is important to provide plots of the data and model estimates in ways that allow referees and readers to evaluate findings, also when complex Bayesian models are employed.

## Supplementary Material

R script for analyses

## Data accessibility

This article has no additional data.
