# Supplementary material for: Blood may buy goodwill: no evidence for a positive relationship between legal culling and poaching in Wisconsin
Source: Proc Biol Sci. 2017 Nov 22;284(1867):20170267. doi: 10.1098/rspb.2017.0267 (PMC5719162; doi:10.1098/rspb.2017.0267)
Supplement: R script for analyses [file rspb20170267supp1.pdf]

```
##############################
# Read in data
##############################


load(file="chapron_treves_2016.Rdata")   # Obtained from Chapron, G. & Treves, A. 2016 Data from: Blood does not buy goodwill: allowing culling increases poaching of
a large carnivore.
                                         # Dryad Data Repository. (doi:10.5061/dryad.b7d7v).
require(gplots)
require(rjags)


pop.data <- as.data.frame(matrix(c(                # Corrected wolf population data from Chapron, G. & Treves, A. 2016 Correction to 'Blood does not buy goodwill:
1995, 83,  86,  0,  80,  0,  0,   0,               # allowing culling increases poaching of a large carnivore'.
1996, 99,  105, 0,  116, 0,  0,   0,               # Proceedings of the Royal Society B: Biological Sciences 283. (doi:10.1098/rspb.2016.2577)
1997, 148, 151, 0,  113, 0,  0,   0,
1998, 178, 184, 0,  139, 0,  0,   0,
1999, 205, 211, 1,  169, 0,  0,   0,
2000, 248, 259, 0,  216, 0,  0,   0,
2001, 257, 259, 0,  249, 0,  0,   0,
2002, 327, 343, 0,  278, 0,  13,  13,
2003, 335, 353, 18, 321, 1,  365, 365,
2004, 373, 410, 25, 360, 9,  303, 303,
2005, 435, 465, 33, 405, 3,  151, 151,
2006, 467, 504, 22, 434, 10, 131, 119,
2007, 540, 577, 35, 509, 19, 365, 365,
2008, 537, 564, 43, 520, 5,  166, 166,
2009, 626, 662, 11, 577, 4,  57,  57,
2010, 690, 733, 18, 557, 5,  0,   0,
2011, 782, 824, 16, 687, 10, 78,  78,
2012, 815, 880, NA, NA,  NA, NA,  NA), nrow=18, ncol=8, byrow=T))

colnames(pop.data) <- c("year", "WI.min", "WI.max", "WI.H", "MI.min", "MI.H", "D.WI.cull", "D.MI.cull")


pop.data<-merge(pop.data, area, all.x=T)


##################################################################################################################################################################
###
#
# Analysis of relationship between total area used and wolf population size
#
##################################################################################################################################################################
###

#####################################################################
# log area by wolf population size
#####################################################################

sink("log.area.bug")
cat("
model{
   # observation model
for (i in 1:nyears){
    ones[i] ~ dinterval(WI.N[i] , Nlim[i,])
    WI.N[i]~dpois(mu[i])
```

```
      }
# process model
for (j in 1:nyears){
    pred.logarea[j]=beta.larea[1]+beta.larea[2]*WI.N[j]
    log.area[j]~dnorm(pred.logarea[j], sigma.larea)
       }
# Priors
for( k in 1:2) {beta.larea[k]~dnorm(0,0.001)}
sigma.larea~dgamma(0.001,0.001)
for (i in 1:nyears){
  mu[i]~dunif(Nlim[i,1],Nlim[i,2])
          }
    }
",fill = TRUE)
sink()

#########################################################################
# PREPARE FOR MCMC
#########################################################################


pop.data.area<-pop.data[!is.na(pop.data$area),]
nyears <- nrow(pop.data.area)

bugs.data <- list(
      nyears = nyears,
      Nlim=cbind(pop.data.area$WI.min,pop.data.area$WI.max),
  log.area = log(pop.data.area$area),
#  area = pop.data.area$area,
  ones=rep(1, nyears)
)

bugs.monitor <- c(
      "WI.N",
#     "beta.area",
      "beta.larea",
#  "sigma.area",
  "sigma.larea",
  "pred.logarea"
#   "pred.area"
)

bugs.chains <- 8



#########################################################################
# MCMC
#########################################################################


logarea.mcmc <- jags.model("log.area.bug", data=bugs.data,n.chains = bugs.chains, n.adapt = 50000)            # set up jags model
wolf.logarea <- coda.samples(model=logarea.mcmc,variable.names=bugs.monitor,50000, thin=100)
summary(wolf.logarea)
gelman.diag(wolf.logarea)
heidel.diag(wolf.logarea)

#########################################################################
# Polynomial area - population size model
```

```
########################################################################
sink("area.bug")
cat("
model{
   # observation model
for (i in 1:nyears){
    ones[i] ~ dinterval(WI.N[i] , Nlim[i,])
    WI.N[i]~dpois(mu[i])
      }
# process model
for (j in 1:nyears){
    pred.area[j]=beta.area[1]*WI.N[j]+beta.area[2]*WI.N[j]*WI.N[j]
    area[j]~dnorm(pred.area[j], sigma.area)
       }
# Priors
for( k in 1:2) {beta.area[k]~dnorm(0,0.001)}
sigma.area~dgamma(0.001,0.001)
for (i in 1:nyears){
  mu[i]~dunif(Nlim[i,1],Nlim[i,2])
          }
    }
",fill = TRUE)
sink()


########################################################################
# PREPARE FOR MCMC
########################################################################


pop.data.area<-pop.data[!is.na(pop.data$area),]
nyears <- nrow(pop.data.area)

bugs.data <- list(
      nyears = nyears,
      Nlim=cbind(pop.data.area$WI.min,pop.data.area$WI.max),
#  log.area = log(pop.data.area$area),
  area = pop.data.area$area,
  ones=rep(1, nyears)
)

bugs.monitor <- c(
      "WI.N",
      "beta.area",
#     "beta.larea",
  "sigma.area",
#  "sigma.larea",
#  "pred.logarea"
   "pred.area"
)

bugs.chains <- 8


########################################################################
# MCMC
########################################################################
```

```
area.mcmc <- jags.model("area.bug", data=bugs.data,n.chains = bugs.chains, n.adapt = 50000)
wolf.area <- coda.samples(model=area.mcmc,variable.names=bugs.monitor,50000, thin=100)
summary(wolf.area)
gelman.diag(wolf.area)
heidel.diag(wolf.area)

########################################################################
# Figure 1
########################################################################


area1.est<-summary(wolf.logarea)
rnam.area1<-rownames(area1.est[[1]])

area2.est<-summary(wolf.area)
rnam.area2<-rownames(area2.est[[1]])

# Figure 1. Plot of area occupied by wolves against wolf population size
plot((pop.data$WI.min+pop.data$WI.max)/2, pop.data$area, pch=15, xlab="Wolf population size", ylab="Area used by wolf packs", type="n", cex.lab=1.5, cex.axis=1.5,
cex=0.5, ylim=c(20000, 67000), xlim=c(200, 850))
plotCI((pop.data$WI.min+pop.data$WI.max)/2, y = pop.data$area, ui=pop.data$WI.max , li=pop.data$WI.min ,add=T, err="x", lwd=2, col=0, barcol="black", gap=0,
sfrac=0.005)

lines(area1.est[[1]][grep("^WI.N", rnam.area1),1], exp(area1.est[[1]][grep("^pred.logarea", rnam.area1),1]), lwd=3)
lines(area2.est[[1]][grep("^WI.N", rnam.area2),1], area2.est[[1]][grep("^pred.area", rnam.area2),1], lty=2, lwd=3)

dev.copy(tiff, filename = "Fig_1.tif", compression=c("lzw"), units="cm", width=16, height=16, res=600)
dev.off()

######################################################################################################################################################################
#####################################
# End of analysis of relationship between total area used and wolf population size
######################################################################################################################################################################
#####################################


######################################################################################################################################################################
###
#
# Fitting model with slope estimates for the relationship between legal state culling and wolf population growth that are allowed to differ between the two states
#
######################################################################################################################################################################
###


#########################################################################
# jags model
#########################################################################

sink("state.bug")
cat("

model {

	##################################
	# Priors
	##################################
```

```
        tauProc <- 1/sigmaProc^2
        sigmaProc ~ dunif(0, 0.5)

        scale.min ~ dnorm(1, 1.0E-6)T(0, 1)
        scale.max ~ dnorm(1, 1.0E-6)T(1, 10)

        sigmaObs ~ dunif(0, 100)
        sigmaObs.Max ~ dunif(0, 100)

        WI.beta.growth.0 ~ dnorm(0, 1.0E-6)
        MI.beta.growth.0 ~ dnorm(0, 1.0E-6)
        beta.growth.wi ~ dnorm(0, 1.0E-6)
beta.growth.mi ~ dnorm(0, 1.0E-6)

        compens ~ dnorm(1.06, 1/0.0714^2)

        WI.N[1] ~ dgamma(1.0E-6, 1.0E-6)
        MI.N[1] ~ dgamma(1.0E-6, 1.0E-6)

        ##################################
        # Process model
        ##################################

        for (t in 2:nyears) {

              WI.growth[t-1] <- WI.beta.growth.0 + beta.growth.wi*WI.treat[t-1]
              WI.Nproc[t-1] <- log( max( 1, WI.N[t-1] * exp(WI.growth[t-1]) - compens*WI.H[t-1] ) )
              WI.N[t] ~ dlnorm(WI.Nproc[t-1], tauProc)

        }

        for (t in 2:(nyears-1)) {

              MI.growth[t-1] <- MI.beta.growth.0 + beta.growth.mi*MI.treat[t-1]
              MI.Nproc[t-1] <- log( max( 1, MI.N[t-1] * exp(MI.growth[t-1]) - compens*MI.H[t-1] ) )
              MI.N[t] ~ dlnorm(MI.Nproc[t-1], tauProc)

        }

        ##################################
        # Observation model
        ##################################

        for (t in 1:nyears) {

              WI.shapeObs[t] <- WI.N[t]*WI.N[t]/(sigmaObs*sigmaObs)
              WI.rateObs[t] <- WI.N[t]/(sigmaObs*sigmaObs)
              WI.gamNobs[t] ~ dgamma(WI.shapeObs[t], WI.rateObs[t])
              WI.Nobs.Min[t] ~ dpois(scale.min*WI.gamNobs[t])

              WI.shapeObs.Max[t] <- WI.N[t]*WI.N[t]/(sigmaObs.Max*sigmaObs.Max)
              WI.rateObs.Max[t] <- WI.N[t]/(sigmaObs.Max*sigmaObs.Max)
              WI.gamNobs.Max[t] ~ dgamma(WI.shapeObs.Max[t], WI.rateObs.Max[t])
              WI.Nobs.Max[t] ~ dpois(scale.max*WI.gamNobs.Max[t])

        }

        for (t in 1:(nyears-1)) {
```

```
				MI.shapeObs[t] <- MI.N[t]*MI.N[t]/(sigmaObs*sigmaObs)
				MI.rateObs[t] <- MI.N[t]/(sigmaObs*sigmaObs)
				MI.gamNobs[t] ~ dgamma(MI.shapeObs[t], MI.rateObs[t])
				MI.Nobs[t] ~ dpois(scale.min*MI.gamNobs[t])

			}

		}

",fill = TRUE)
sink()

##########################################################################
# PREPARE FOR MCMC
##########################################################################

nyears <- nrow(pop.data)

bugs.data <- list(
		nyears = nyears,
		WI.Nobs.Min=pop.data$WI.min,
		WI.Nobs.Max = pop.data$WI.max,
		WI.H = pop.data$WI.H,
		WI.treat = pop.data$D.WI.cull/365,

		MI.Nobs = pop.data$MI.min[-nyears],
		MI.H = pop.data$MI.H[-nyears],
		MI.treat = pop.data$D.MI.cull[-nyears]/365
)

bugs.monitor <- c(
		"sigmaProc",
		"compens",
		"scale.min",
		"scale.max",
		"sigmaObs",
		"sigmaObs.Max",
		"WI.beta.growth.0",
		"MI.beta.growth.0",
		"beta.growth.wi",
		"beta.growth.mi",
		"WI.N",
		"MI.N"
)

bugs.chains <- 8

bugs.inits <- function(){
		list(
			WI.N = runif(nyears, 0.5, 1.5)*round((pop.data$WI.min + pop.data$WI.max)/2),
			MI.N = runif(nyears-1, 0.5, 1.5)*pop.data$MI.min[-nyears]
		)
}

##########################################################################
# MCMC
##########################################################################
```

```
bugs.mcmc2<-jags.model("state.bug", data =bugs.data,inits=bugs.inits, n.chains = bugs.chains, n.adapt = 100000)
wolf.mcmc2 <- coda.samples(model=bugs.mcmc2,variable.names=bugs.monitor,n.iter=100000,thin=20) #
gelman.diag(wolf.mcmc2)
heidel.diag(wolf.mcmc2)

summary(wolf.mcmc2)


#######################################################################################################################################################################
#####################################
# End of estimation of the effect of legal state culling on wolf population growth in the two states
#######################################################################################################################################################################
#####################################



#######################################################################################################################################################################
###
#
# Analysis of variation in probability of reproduction in Wisconsin
#
#######################################################################################################################################################################
###


pop.data$pdays.W<-pop.data$D.WI.cull/365
pop.data$pdays.M<-pop.data$D.MI.cull/365
packs_t<-merge(packs, pop.data, all.x=T)

cor.test(pop.data$pdays.W, pop.data$WI.H)

####################################################################################################################
# Estimation of annual probabilities of reproduction
####################################################################################################################


library(lme4)
lme.breed.D<-glmer(did_bred1~pdays.W+(1|year), data=packs_t, family=binomial)
anova(lme.breed.D)
summary(lme.breed.D)

lme.breed.H<-glmer(did_bred1~WI.H+(1|year), data=packs_t, family=binomial)
anova(lme.breed.H)
summary(lme.breed.H)

lme.breed.DH<-glmer(did_bred1~WI.H+pdays.W+(1|year), data=packs_t, family=binomial)
anova(lme.breed.DH)
summary(lme.breed.DH)


est.breed_year<-glm(did_bred1~-1+factor(year), data=packs, family=binomial)
summary(est.breed_year)

breed<-data.frame(year=1995:2011, breed.p=plogis(summary(est.breed_year)$coefficients[,1]))
pop.data<-merge(pop.data, breed, all.x=T)


cor.test(pop.data$breed.p, pop.data$pdays.W)
```

```
cor.test(pop.data$breed.p, pop.data$WI.H)
cor.test(pop.data$pdays.W, pop.data$WI.H)

# Plot of probability of reproduction by legal state culling
plot(pop.data$pdays.W[-18], plogis(summary(est.breed_year)$coefficients[,1]), ylim=c(0,1), ylab="Probability of reproduction", xlab="Proportion of year with legal
state culling", pch=16, cex=2, las=1, cex.lab=1.5, cex.axis=1.5)
plotCI(x= pop.data$pdays.W[-18], y = plogis(summary(est.breed_year)$coefficients[,1]), ui=
plogis(summary(est.breed_year)$coefficients[,1]+summary(est.breed_year)$coefficients[,2]), li=plogis(summary(est.breed_year)$coefficients[,1]-
summary(est.breed_year)$coefficients[,2]),add=T, lwd=1, col=0, barcol="black", gap=0.5, sfrac=0.01)
lines(seq(0,1,by=0.05), plogis(summary(lme.breed.D)$coefficients[1,1]+summary(lme.breed.D)$coefficients[2,1]*seq(0,1,by=0.05)), lwd=2)

dev.copy(tiff, filename = "Fig2.tif", compression=c("lzw"), units="cm", width=16, height=16, res=600)
dev.off()



##################################################################################################################################################################
###
#
# Fitting model with that include effect of probability of reproduction on wolf population growth in Wisconsin
#
##################################################################################################################################################################
###



#########################################################################
# BUGS MODEL
#########################################################################

sink("model.rep")
cat("

model {

           ##################################
           # Priors
           ##################################

           tauProc <- 1/sigmaProc^2
           sigmaProc ~ dunif(0, 0.5)

           scale.min ~ dnorm(1, 1.0E-6)T(0, 1)
           scale.max ~ dnorm(1, 1.0E-6)T(1, 10)

           sigmaObs ~ dunif(0, 100)
           sigmaObs.Max ~ dunif(0, 100)

           WI.beta.growth.0 ~ dnorm(0, 1.0E-6)
           MI.beta.growth.0 ~ dnorm(0, 1.0E-6)
           beta.growth.wi ~ dnorm(0, 1.0E-6)
    beta.growth.mi ~ dnorm(0, 1.0E-6)
    beta.growth.rep.wi ~ dnorm(0, 1.0E-6)

           compens ~ dnorm(1.06, 1/0.0714^2)

           WI.N[1] ~ dgamma(1.0E-6, 1.0E-6)
           MI.N[1] ~ dgamma(1.0E-6, 1.0E-6)
```

```
        ##################################
        # Process model
        ##################################

        for (t in 2:nyears) {

             WI.growth[t-1] <- WI.beta.growth.0 + beta.growth.wi*WI.treat[t-1] + beta.growth.rep.wi * WI.rep[t]
             WI.Nproc[t-1] <- log( max( 1, WI.N[t-1] * exp(WI.growth[t-1]) - compens*WI.H[t-1] ) )
             WI.N[t] ~ dlnorm(WI.Nproc[t-1], tauProc)

        }

        for (t in 2:(nyears)) {

             MI.growth[t-1] <- MI.beta.growth.0 + beta.growth.mi*MI.treat[t-1]
             MI.Nproc[t-1] <- log( max( 1, MI.N[t-1] * exp(MI.growth[t-1]) - compens*MI.H[t-1] ) )
             MI.N[t] ~ dlnorm(MI.Nproc[t-1], tauProc)

        }

        ##################################
        # Observation model
        ##################################

        for (t in 1:nyears) {

             WI.shapeObs[t] <- WI.N[t]*WI.N[t]/(sigmaObs*sigmaObs)
             WI.rateObs[t] <- WI.N[t]/(sigmaObs*sigmaObs)
             WI.gamNobs[t] ~ dgamma(WI.shapeObs[t], WI.rateObs[t])
             WI.Nobs.Min[t] ~ dpois(scale.min*WI.gamNobs[t])

             WI.shapeObs.Max[t] <- WI.N[t]*WI.N[t]/(sigmaObs.Max*sigmaObs.Max)
             WI.rateObs.Max[t] <- WI.N[t]/(sigmaObs.Max*sigmaObs.Max)
             WI.gamNobs.Max[t] ~ dgamma(WI.shapeObs.Max[t], WI.rateObs.Max[t])
             WI.Nobs.Max[t] ~ dpois(scale.max*WI.gamNobs.Max[t])

        }

        for (t in 1:(nyears-1)) {

             MI.shapeObs[t] <- MI.N[t]*MI.N[t]/(sigmaObs*sigmaObs)
             MI.rateObs[t] <- MI.N[t]/(sigmaObs*sigmaObs)
             MI.gamNobs[t] ~ dgamma(MI.shapeObs[t], MI.rateObs[t])
             MI.Nobs[t] ~ dpois(scale.min*MI.gamNobs[t])

        }

   }

",fill = TRUE)
sink()

#########################################################################
# PREPARE FOR MCMC
#########################################################################

nyears <- nrow(pop.data)

bugs.data <- list(
```

```
      nyears = nyears-1,
      WI.Nobs.Min=pop.data$WI.min[-nyears],
      WI.Nobs.Max = pop.data$WI.max[-nyears],
      WI.H = pop.data$WI.H[-nyears],
      WI.treat = pop.data$D.WI.cull[-nyears]/365,
  WI.rep = pop.data$breed.p[-nyears],
      MI.Nobs = pop.data$MI.min[-nyears],
      MI.H = pop.data$MI.H[-nyears],
      MI.treat = pop.data$D.MI.cull[-nyears]/365
)

bugs.monitor <- c(
      "sigmaProc",
      "compens",
      "scale.min",
      "scale.max",
      "sigmaObs",
      "sigmaObs.Max",
      "WI.beta.growth.0",
      "MI.beta.growth.0",
      "beta.growth.wi",
      "beta.growth.mi",
      "beta.growth.rep.wi",
      "WI.N",
      "MI.N"
)

bugs.chains <- 8

bugs.inits <- function(){
      list(
            WI.N = runif(nyears-1, 0.5, 1.5)*round((pop.data$WI.min[-nyears] + pop.data$WI.max[-nyears])/2),
            MI.N = runif(nyears-1, 0.5, 1.5)*pop.data$MI.min[-nyears]
      )
}

##########################################################################
# MCMC
##########################################################################


bugs.rep<-jags.model("model.rep", data =bugs.data,inits=bugs.inits, n.chains = bugs.chains, n.adapt = 100000)
wolf.rep <- coda.samples(model=bugs.rep,variable.names=bugs.monitor,n.iter=100000,thin=20) #
gelman.diag(wolf.rep)                                                                                    # calculate Gelman-Rubin statistics to evaluate convergence
heidel.diag(wolf.rep)

summary(wolf.rep)

########
# End
########
```
